# Comprehensive Optical Coherence Tomography–Guided RotaTripsy for Massive Coronary Calcified Nodule: A Decision-Making Approach

**DOI:** 10.1016/j.jaccas.2024.102566

**Published:** 2024-10-02

**Authors:** Keisuke Yasumura, Manish Vinayak, Amit Hooda, Samin K. Sharma, Annapoorna S. Kini

**Affiliations:** a Department of Cardiology, The Zena and Michael A. Wiener Cardiovascular Institute, Icahn School of Medicine at Mount Sinai, New York, New York, USA

**Keywords:** intravascular imaging, intravascular lithotripsy, rotational atherectomy

## Abstract

**Objectives:**

We aim to describe the step-by-step optical coherence tomography–guided rotational atherectomy and intravascular lithotripsy for treating substantial coronary calcified nodules.

**Key Steps:**

These include initial rotational atherectomy with a 1.5-mm burr, multiple optical coherence tomography imaging studies to assess lesion morphology, upsizing the rotational burr to 2.0 mm for further debulking, performing adjunctive intravascular lithotripsy with a 3.5-mm Shockwave balloon (Shockwave Medical Inc), and final stent deployment and optimization.

**Potential Pitfalls:**

Potential complications include burr entrapment during rotational atherectomy, inadequate lesion modification with initial rotational atherectomy, and failure to achieve deep calcium fractures leading to stent underexpansion. To avoid these complications, start with a smaller burr size and use a slow pecking motion, conduct a meticulous intravascular imaging assessment, and upsize the burr and adjunctive intravascular lithotripsy on the basis of intravascular imaging findings.

**Take-Home Messages:**

Detailed intravascular imaging is crucial for guiding personalized treatment strategies. Combining rotational atherectomy and intravascular lithotripsy (RotaTripsy) provides a synergistic approach for treating extensive calcified nodules.

## Graphical Abstract

**Figure:**
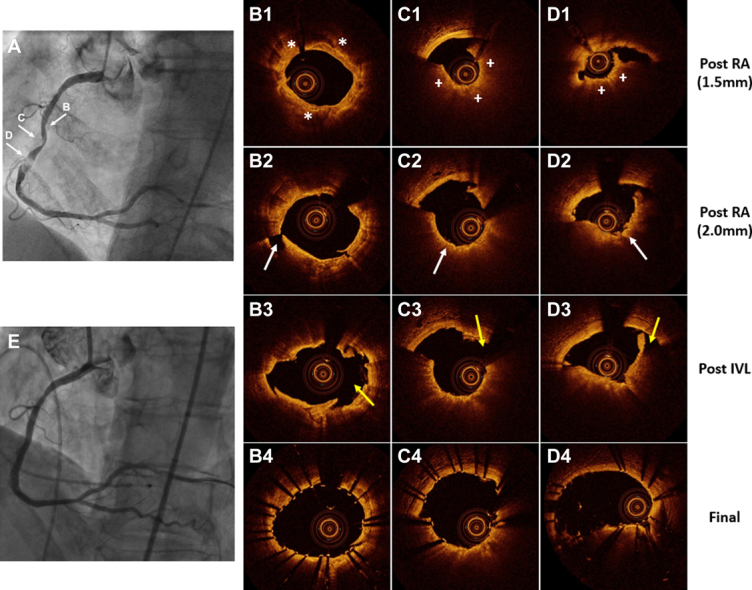

Coronary calcified nodules (CN) pose significant challenges in cardiovascular interventions, by necessitating precise assessment and targeted treatment strategies.^1^ CNs represent a complex aspect of coronary artery calcification, often coexisting with extensive calcified sheets proximally or distally to the CN site.^2^ On the basis of high-resolution intravascular imaging, such as optical coherence tomography (OCT), CNs are classified into eruptive and noneruptive types, depending on whether there is disruption of the superficial fibrous cap.^3^ Although CNs are recognized as a rare but potential cause of acute coronary syndrome,^4^^,^^5^ specific treatment algorithms or detailed recommendations remain sparse in current expert consensus.^6^^,^^7^ Addressing these lesions requires techniques that not only ablate the surface of the CN but also create fractures in the surrounding calcified sheets to ensure optimal stent expansion.^8^ The combination of rotational atherectomy (RA) with intravascular lithotripsy (IVL), termed RotaTripsy,^9^ offers a synergetic approach for treating such intricate calcified lesions associated with CNs. This technique integrates the mechanical debulking capability of RA with the acoustic pulse-wave technology of IVL. Here we present a case with step-by-step OCT-guided RA and IVL for treating substantial CNs.

**Take-Home Messages**

• Use comprehensive intravascular imaging to assess and characterize coronary CNs accurately, thus facilitating effective treatment planning.
• Select appropriate interventional devices on the basis of detailed plaque morphology, by using step-by-step OCT guidance to optimize treatment outcomes for complex calcified coronary lesions involving extensive CNs.

## Case Summary

An 81-year-old woman with known risk factors of hypertension, hyperlipidemia, non–insulin-dependent diabetes mellitus, and a past history of smoking presented with progressively worsening angina of Canadian Cardiovascular Society class III severity for the past 3 months despite optimal medical therapy. In addition, she had a history of paroxysmal atrial fibrillation (treated with anticoagulation), hypothyroidism, bronchiolitis obliterans, and breast cancer. Her vital signs on arrival were normal, with a blood pressure of 129/75 mm Hg and a heart rate of 65 beats/min. Physical examination was unremarkable. The cardiac enzymes were not elevated. Her electrocardiogram showed atrial fibrillation with nonspecific ST-segment changes. Transthoracic echocardiography showed a normal left ventricular ejection fraction of 60% with no regional wall motion abnormalities. Coronary computed tomography angiography revealed severe 2-vessel disease involving the right coronary artery (RCA) and the left circumflex (LCx) artery, with a notably high coronary calcium score of 4,320. Specifically, the calcium score was 3,204 for the RCA, 206 for the LCx artery, and 910 for the left anterior descending artery. Computed tomography–derived fractional flow reserve indicated significant flow limitations, with values of 0.63 in the RCA and 0.74 in the left circumflex artery. The patient was managed with amlodipine, atorvastatin, hydrochlorothiazide, levothyroxine, anastrozole, metformin, and apixaban, and clopidogrel was initiated on admission. She was transferred to the catheterization laboratory for diagnostic angiography and potential percutaneous coronary intervention (PCI).

## Procedural Steps

**Figure 1:**
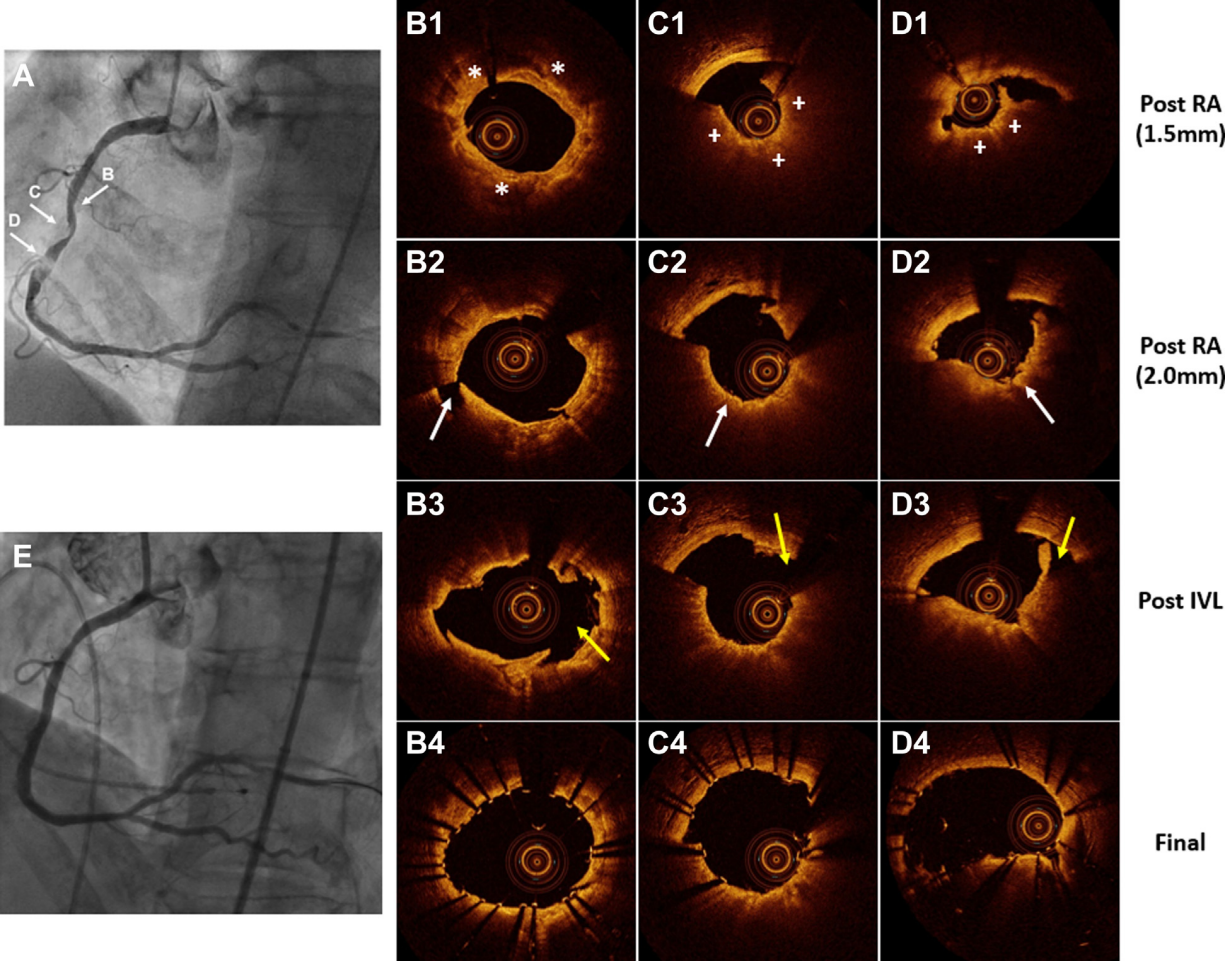
Optical Coherence Imaging–Guided RotaTripsy for Extensive Calcified Nodules in the Right Coronary Artery (A) Initial diagnostic coronary angiography of the severely calcified right coronary artery lesion. Arrows B to D indicate corresponding optical coherence tomography cross-sections. (B1 to D1) Post-1.5-mm rotational atherectomy (RA) optical coherence tomography images; (B1) shows a 360° calcified plaque (asterisks), (C1) shows an ablated 270° of calcified nodule (plus signs), and (D1) shows an obstructive calcified nodule (plus signs). (B2 to D2) Post-2.0-mm rotational atherectomy optical coherence tomography images; (B2) shows small calcium fracture (white arrow), (C2) shows more extensive ablation of a calcified nodule (white arrow), and (D2) shows an ablated obstructive calcified nodule (white arrow). (B3 to D3) Post-intravascular lithotripsy (IVL) optical coherence tomography images showing large calcium fractures (yellow arrows). (B4 to D4) The final poststent optical coherence tomography images demonstrating well-expanded stent cross-sections. (E) Final angiography.

Coronary angiography revealed a normal left main artery with mild disease in the left anterior descending artery and severely stenotic tandem lesions in the LCx artery. The RCA was a dominant vessel with a severely stenotic, heavily calcified lesion that exhibited a hazy and substantial filling defect in the mid-RCA while maintaining TIMI flow grade 3 (Figure 1A, Video 1). Given the anatomical complexity and comorbidities, the patient underwent PCI of the LCx artery, followed by staged revascularization of the RCA.

Because of the severe stenosis and extensive calcification noted on angiography, the RCA lesion was potentially uncrossable by an intravascular imaging catheter. To address this, upfront RA using a 1.5-mm burr of RotaPro (Boston Scientific) was performed to modify the heavily calcified obstruction. Subsequent OCT interrogation with a Dragonfly imaging catheter (Abbott Vascular) revealed multiple obstructive CNs accompanied by substantial adjacent calcified plaque (Figures 1B1 to 1D1, Video 2). Despite the initial rotational ablation, significant stenosis secondary to the CN with the minimum lumen diameter of 0.9 mm was noted, and no major modifications were observed in the adjacent calcified sheet. Consequently, the decision was made to escalate to upsize the burr to 2.0 mm to further debulk the calcified lesion. Post-2.0-mm RA OCT confirmed more extensive ablation of the CNs with the minimal lumen diameter of 1.96 mm, and minimal fractures observed in the thick calcified sheet, which measured up to 1.2 mm in thickness (Figures 1B2 to 1D2, Video 3). Given the thick, calcified, potentially balloon-undilatable lesion, we decided to attempt adjunctive IVL with a 3.5-mm Shockwave C2+ balloon (Shockwave Medical Inc) at 4 to 6 atm for 120 pulses. Post-IVL OCT imaging demonstrated further modifications in the CNs, and deep calcium fractures were noted within the circumferential calcified sheet (Figure 1B3 to 1D3, Video 4). The procedure was concluded with the successful deployment of a 4.0 mm × 48 mm Synergy XD (Boston Scientific) drug-eluting stent followed by adequate post-dilation, resulting in satisfactory stent expansion with a final minimal stent area of 7.0 mm^2^, without major stent edge dissection or malapposition (Figures 1B4 to 1D4, Video 5), as well as maintaining TIMI flow grade 3, as confirmed by angiography (Figure 1E, Video 6). The patient was discharged the day after the procedure without any adverse events and remained asymptomatic on subsequent 3-month follow-up.

## Potential Pitfalls

In the treatment of CNs, contemporary PCI poses significant challenges, often resulting in poor long-term outcomes. Eruptive and noneruptive CNs have distinct histopathologic and prognostic features. Intravascular imaging plays a vital role in differentiating these subtypes, and this is crucial for developing optimal treatment strategies. While we await the results of ongoing studies, a tailored therapeutic approach that is based on the specific features of different CNs is recommended.^10^

During the procedure for complex calcified lesions with CNs, several potential complications may arise that need to be carefully managed to ensure successful outcomes. One significant risk is burr entrapment during RA. This can occur if the burr becomes stuck within the obstructive CN, which can lead to vessel injury, perforation, or prolonged procedural time. Entrapment can also increase the likelihood of adverse outcomes. Another issue is inadequate lesion modification with the initial RA. In case of an extremely dense CN, the initial RA may not sufficiently modify the lesion, thus making subsequent interventions, such as stent deployment, challenging. This can result in suboptimal lesion preparation and incomplete revascularization, potentially leading to procedural failure. Failure to achieve calcium fractures is another critical pitfall that can result in stent underexpansion. Deep calcium fractures are essential for optimal stent expansion because they allow the stent to fully expand and appose to the vessel wall. Without these fractures, there is an increased risk of stent thrombosis, restenosis, and other adverse clinical outcomes.

To mitigate these risks, several strategies can be used. Starting with a smaller burr size and using a slow pecking motion can reduce the risk of burr entrapment. This technique allows gradual advancement through the calcified plaque, thereby minimizing abrupt resistance and ensuring controlled lesion modification. Conducting meticulous intravascular imaging assessment is also crucial. This allows for precise assessment of the extent and nature of calcification, to guide appropriate calcium modification device selection and confirm adequate lesion modification. In cases where initial RA does not achieve sufficient lesion modification, upsizing the burr can be necessary for further debulking of the CN. Additionally, adjunctive IVL can be used to achieve deeper calcium fractures, particularly in balloon-undilatable lesions. By understanding and addressing these potential pitfalls through careful planning and execution, clinicians can enhance the safety and efficacy of the procedure and ultimately improve patient outcomes in the management of complex calcified coronary lesions with extensive CNs. Although we could have contemplated using high-pressure inflation with a noncompliant balloon to confirm vessel compliance after the initial RA runs, intravascular imaging clearly showed minimal fractures in the flanking thick calcified sheets, thus prompting the use of IVL for further lesion modification. Although a multimodality approach using imaging guidance and the combination of RA and IVL offers a potentially synergistic calcium-modification strategy, evaluating the cost-effectiveness of this dual preparation strategy remains crucial for future research and clinical application. Finally, considering the unique mechanism of poor outcomes of CN PCI resulting from eruptive CN reprotrusion, the role of a stentless revascularization strategy using drug-coated balloons (DCBs) remains unexplored. However, the potential advantage of using DCBs may be nullified by the risk of recoil and regrowth of the CNs.

## Conclusions

This case demonstrates the strategic integration of advanced intravascular imaging and cutting-edge interventional techniques—specifically, the combination of RA and IVL—in managing complex calcified coronary lesions associated with extensive CNs. It emphasizes the importance of a tailored approach, using detailed anatomical insights provided by intravascular imaging, to optimize clinical outcomes. This comprehensive OCT-guided RotaTripsy exemplifies precise decision making and intervention, thereby establishing a standard for addressing challenging CNs.

## Funding Support and Author Disclosures

The authors have reported that they have no relationships relevant to the contents of this paper to disclose.

## Appendix

Video 1 Initial Angiography

Video 2 Post-1.5-mm RA OCT

Video 3 Post-2.0-mm RA OCT

Video 4 Post-IVL OCT

Video 5 Final OCT

Video 6 Final Angiography
